# Transcriptome Analysis and Development of SSR Molecular Markers in *Glycyrrhiza uralensis* Fisch.

**DOI:** 10.1371/journal.pone.0143017

**Published:** 2015-11-16

**Authors:** Yaling Liu, Pengfei Zhang, Meiling Song, Junling Hou, Mei Qing, Wenquan Wang, Chunsheng Liu

**Affiliations:** 1 School of Chinese Pharmacy, Beijing University of Chinese Medicine, Beijing, 100102, PR China; 2 College of Life Science, Shanxi Agricultural University, Taigu, 030801, Shanxi Province, PR China; 3 School of Inner Mongolia Medical University, Hohhot, 010059, PR China; 4 Institute of Medicinal Plant Development, Chinese Academy of Medical Sciences & Peking Union Medical College, Beijing, 100193, PR China; Chinese Academy of Medical Sciences, Peking Union Medical College, CHINA

## Abstract

Licorice is an important traditional Chinese medicine with clinical and industrial applications. Genetic resources of licorice are insufficient for analysis of molecular biology and genetic functions; as such, transcriptome sequencing must be conducted for functional characterization and development of molecular markers. In this study, transcriptome sequencing on the Illumina HiSeq 2500 sequencing platform generated a total of 5.41 Gb clean data. *De novo* assembly yielded a total of 46,641 unigenes. Comparison analysis using BLAST showed that the annotations of 29,614 unigenes were conserved. Further study revealed 773 genes related to biosynthesis of secondary metabolites of licorice, 40 genes involved in biosynthesis of the terpenoid backbone, and 16 genes associated with biosynthesis of glycyrrhizic acid. Analysis of unigenes larger than 1 Kb with a length of 11,702 nt presented 7,032 simple sequence repeats (SSR). Sixty-four of 69 randomly designed and synthesized SSR pairs were successfully amplified, 33 pairs of primers were polymorphism in in *Glycyrrhiza uralensis* Fisch., *Glycyrrhiza inflata* Bat., *Glycyrrhiza glabra* L. and *Glycyrrhiza pallidiflora Maxim*. This study not only presents the molecular biology data of licorice but also provides a basis for genetic diversity research and molecular marker-assisted breeding of licorice.

## Introduction

Licorice is an important herbal medicine because of its high medicinal value and applications in light and food industries. In the Pharmacopoeia of the People’s Republic of China [1], three species of *Glycyrrhiza*, namely, *G*. *uralensis* Fisch., *G*. *inflata* Bat., and *G*. *glabra* L., are listed as authentic medicinal licorice. Licorice is mainly distributed in China, especially in the northeast and north China, as well as in northwest arid, semi-arid, and desert regions.

The active ingredients of licorice include saponins, polysaccharides, flavonoids, and triterpenes; among these content, total saponins, including glycyrrhizic acid, glycrrhetinic acid, and neoisoliquiritin, exert pharmacological effects, such as protection against hepatotoxicity and anti-inflammatory [2–4]. Although licorice has been widely investigated in chemical and pharmacological fields, the metabolic pathways of the active ingredients of this plant have been rarely studied. In particular, insufficient genome and transcriptome sequencing data complicate research on the metabolic pathway of glycyrrhizic acid [5, 6]. At the genome level, RNA sequencing can be used for gene screening analysis to detect gene expression and differences [7, 8]. This technology has been widely utilized for studies on medicinal plants, such as *Polygonum cuspidatum*, because of its high flux and repeatability, wide detection range, and quantitative accurate characteristics [9, 10].

In this study, the latest HiSeq 2500 platform was used for licorice transcriptome sequencing to completely utilize licorice genes and germplasm resources. The resulting sequence data were assembled and annotated. Genes related to glycyrrhizic acid biosynthesis of secondary metabolites were found. This research not only research glycyrrhizic acid biosynthesis of secondary metabolites, but also provides a basis for gene annotation and discovery. In addition, a large number of molecular markers for simple sequence repeats (SSR) were predicted and developed for licorice. These markers can be used for future studies on gene mapping, linkage map development, genetic diversity analysis, and marker-assisted selection breeding of *G*. *uralensis*.

## Materials and Methods

### 1. Plant materials

Plant material was collected from a 4-year-old fresh healthy *G*. *uralensis* plant grown in a field in Beijing, China (the Beijing University of Chinese Medicine Endangered Medicinal Plant Research and Testing Base). roots, stems, and leaves were immediately stored in liquid nitrogen for analysis. In addition, *Glycyrrhiza uralensis* Fisch., *Glycyrrhiza inflata* Bat., *Glycyrrhiza glabra* L., *Glycyrrhiza pallidiflora Maxim*.were chosen to detect polymorphism of primer pairs.

### 2. RNA isolation and transcriptome sequencing

Total RNA was extracted from the roots, stems, and leaves by using an Ed lai kit (Ed Biological Technology Co., Ltd., Beijing; article number: RN40). Nanodrop, Qubit 2.0, and Agilent 2100 were used to determine RNA purity, concentration, and integrity, respectively. mRNA was purified and enriched from the total RNA by using poly (T) low-adsorption magnetic beads. mRNA was interrupted at a high temperature to select its suitable length. Synthesis was continued to the second cDNA chain to purify cDNA. Finally, the resulting cDNA from the mixture of roots, stems, and leaves (3:1:1) was used to construct the transcriptome sequence library. The cDNA library was enriched through PCR and subjected to Illumina HiSeq 2500 high-throughput sequencing. The transcriptome datasets are available in the NCBI Sequence Read Archive (SRA) under accession number SRX1295883.

### 3. *De novo* sequence assembly

The cDNA library was sequenced using the Illumina HiSeq 2500 system. Raw image data from sequencing were transformed by base calling into raw sequence data and defined as raw reads. Clean data were generated from the raw data through data processing, including removal of low-quality reads and adapter sequences. The clean reads were subjected to *de novo* assembly using the Trinity software to recover full-length transcripts across a broad range of expression levels; this technique presents sensitivity similar to genome alignment methods [11]. Transcriptome assembly was then conducted.

### 4. Annotation of unigenes

For functional annotation of unigenes, the sequences were compared using the following databases: Nr (), Swiss–Prot (http://www.uniprot.org/), gene ontology (GO) (http://www.geneontology.org/), Clusters of Orthologous Groups (COG) (http://www.ncbi.nlm.nih.gov/cog/), Kyoto Encyclopedia of Genes and Genomes (KEGG) (http://www.genome.jp/kegg/), and Non-redundant Nucleotide (Nt) (). Comparison was performed using the BLASTX algorithm set at an E-value ≤ 1e^−5^, with the annotation information of homologous genes in the library.

### 5. Development and detection of SSR molecular markers for licorice

Unigenes larger than 1 Kb were subjected to SSR analysis by using the MISA software (http://pgrc.ipk-gatersleben.de/misa/misa.html). Search criteria included the number of repetitions for mono-, di-, tri-, tetra-, penta-, and hexa-nucleotides, with repetition times of 10, 6, 5, 4, 3, 3, and 2. Primers for each SSR were designed using the Primer 6 software. A total of 69 primer pairs were obtained and used for amplification. Detailed information about the designed primers is shown in S1 Table. DNA for PCR amplification was extracted from different samples through cetrimonium bromide method [12]. PCR amplification was conducted as follows: denaturation at 94°C for 3 min, followed by 35 cycles of 94°C for 40 s, 55°C–60°C for 30 s, and 72°C for 60 s. Final extension was performed at 72°C for 5 min. PCR products were analyzed through electrophoresis on 2.5% agarose gels.

## Results and Analysis

### 1. RNA sequencing and assembly of licorice transcriptome

To obtain transcriptome information of licorice, we extracted the total RNA from the roots, stems, and leaves mixed at 3:1:1 ratio and sequenced through HiSeq 2500. A total of 26,766,870 raw sequencing reads were generated. By removing the adaptors and low-quality data, we obtained 5.41 Gb clean reads, 45.22% GC, and 0.05% N. The base quality value Q30 reached 86.59%, which indicates satisfactory sequencing quality of the licorice samples. The obtained data were used for further analysis.

**Fig 1:**
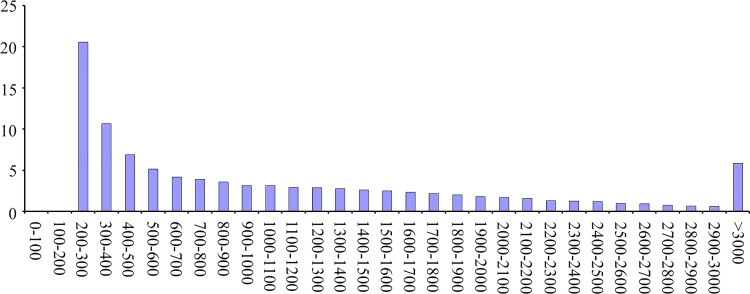
Frequency distribution of transcripts.

**Fig 2:**
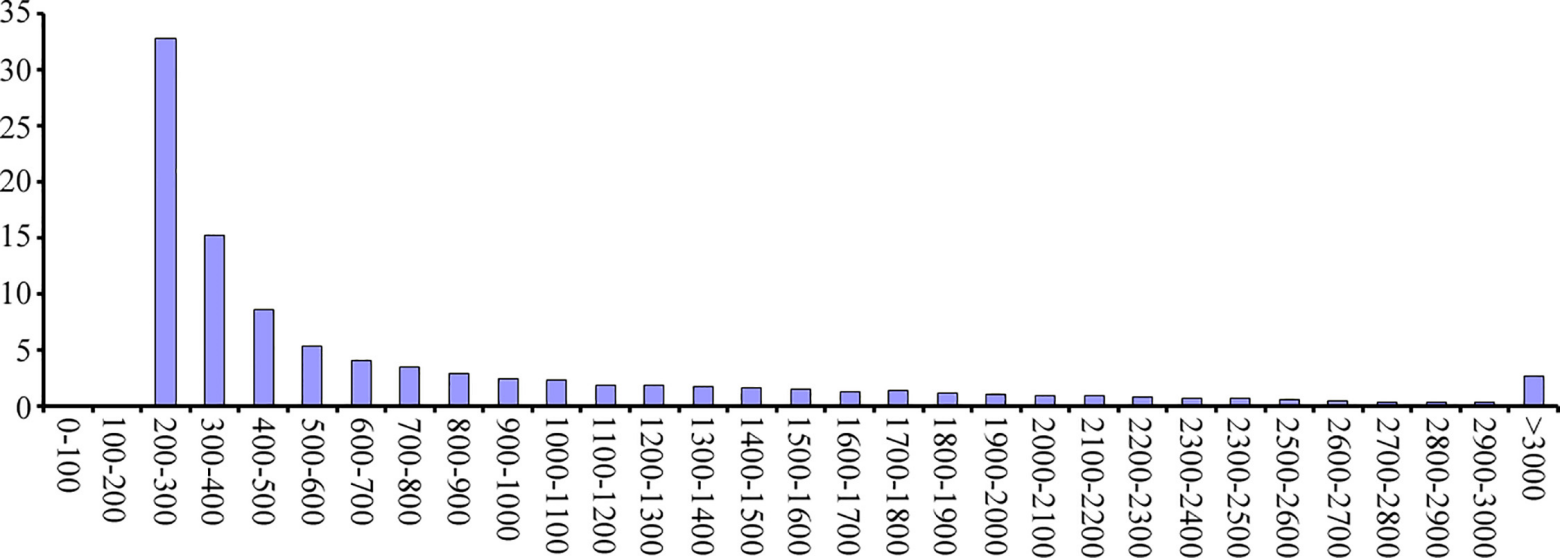
Frequency distribution of unigenes.

Sample data were merged and assembled using the Trinity software. By using the overlapping information in high-quality reads, we obtained 3,114,638 contigs with an average length of 51.00 nt and N50 length of 48 nt (Table 1). The contigs were clustered according to the similarity of the paired-end information and contigs. The clustering yielded 87,242 transcripts with an average length of 1121.92 nt in the assembled part (Table 1). Further assembly generated 46,641 unigenes (total length of 36,725,337 nt and average length of 787.40 nt) (Table 1). For transcripts, the size range of 1000–2000 nt accounted for most about 25.11% of all transcripts, and unigene with lengths 200–300 nt accounted for most about 32.78% of all unigene, the frequency distribution of transcripts and unigenes are shown in Fig 1 and Fig 2, respectively.

**Table 1:** Assembly results.

Length Range (nt)	Contigs	Transcript	Unigene
0–100	2960407 (95.05%)	0	0
100–200	98089 (3.15%)	0	0
200–300	19013 (0.61%)	17,951 (20.58%)	15,287 (32.78%)
300–500	14,149 (0.45%)	15,279 (17.51%)	11,103 (23.81%)
500–1000	10,723 (0.34%)	17,416 (19.96)	8,549 (18.33%)
1000–2000	8,157 (0.26%)	21,904 (25.11%)	7,532 (16.15%)
2000+	4,100 (0.13%)	14,692 (16.84%)	4,170 (8.94%)
Total Number	3,114,638	87,242	46,641
Total Length	158,837,163	97,878,454	36,725,337
N50 Length	48	1,816	1,395
Mean Length	51.00	1121.92	787.40

### 2. Annotation of licorice functional genes

To identify the gene function and GO classification of licorice, we annotated the unigenes through BLAST search against the non-redundant database (Nr/Nt), with a significance threshold of an e-value of 1 × 10^−5^. A total of 29,614 licorice unigenes were obtained through BLAST sequence comparison analysis.

**Fig 3:**
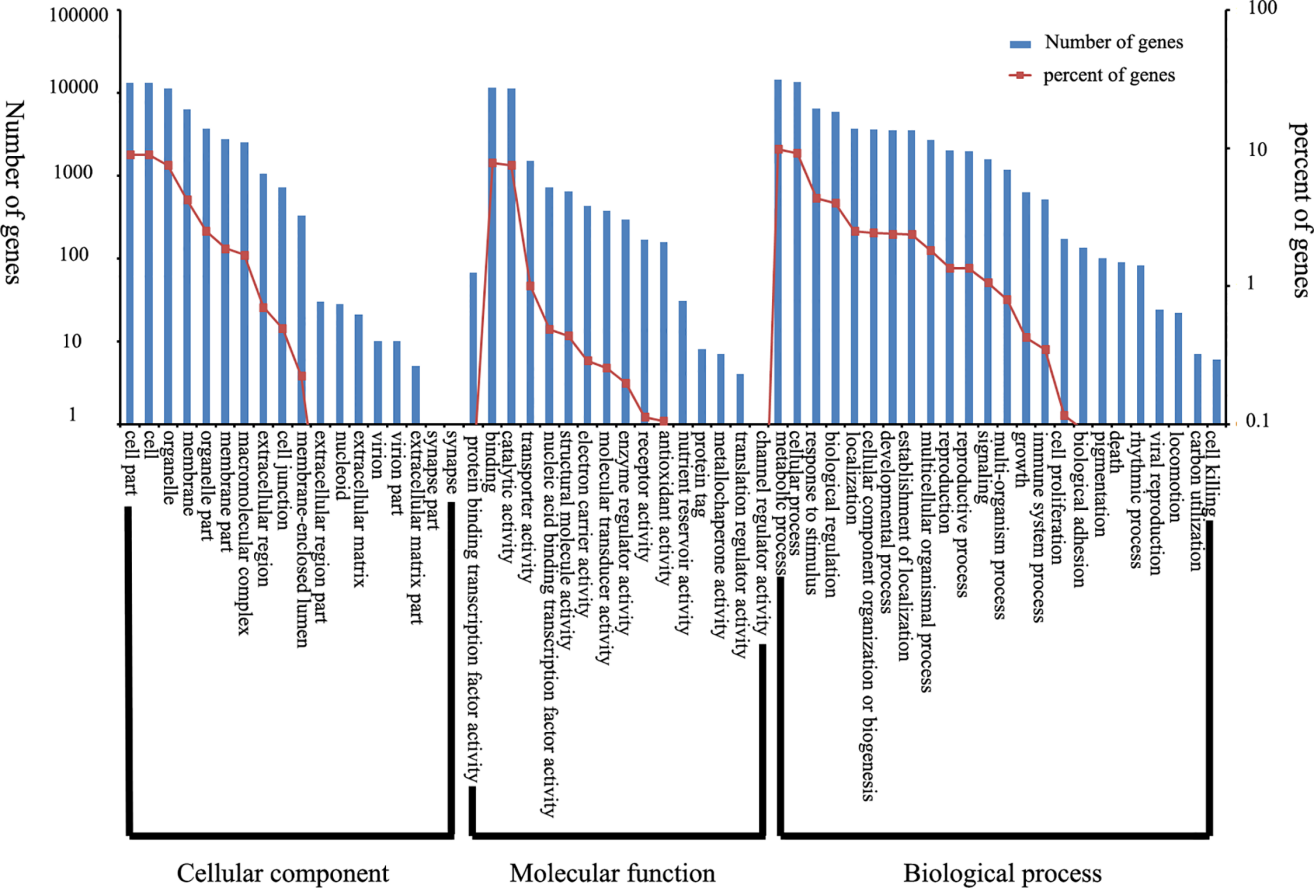
Functional annotation of unigenes based on GO categories.

GO is used to classify gene function and describe the functional attributes of genes and gene products in an organism. Among 29,614 licorice unigenes, 29,389 unigenes were got Nr annotations, accordingly, 22,244 of 29,389 unigenes with Nr annotations were annotated with GO information. The GO classification system comprises three large categories: molecular function, biological process, and cellular components, which can be further divided into 58 small categories (Fig 3). Among all unigenes with GO annotations, 45.59% belong to Biological Process, 22.70% to Cellular Component, and 27.71% to Molecular Function. In Biological Process, oxidation–reduction process (GO: 0055114) accounts for the largest proportion, followed by regulation of transcription, DNA template (GO: 0006355), and protein phosphorylation (GO: 0006468). In Cellular Component, nucleus (GO: 0005634) accounts for the largest proportion, followed by plasma membrane (GO: 0005886) and integral component of membrane (GO: 0016021). In Molecular Function, ATP binding (GO: 0005524) accounts for the largest proportion, followed by zinc-ion binding (GO: 0008270) and DNA binding (GO: 0003677).

**Fig 4:**
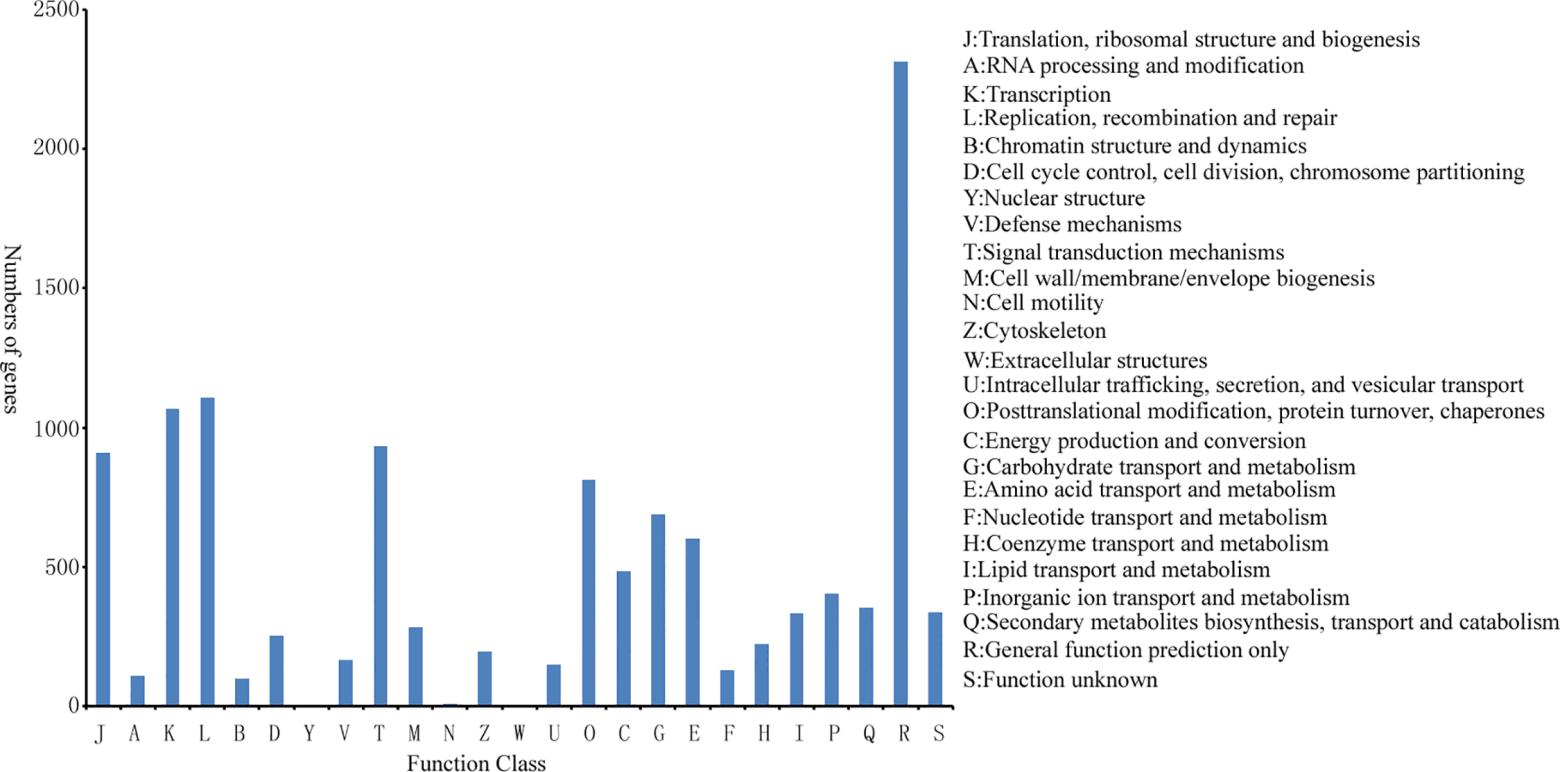
Functional classification of licorice based on COG.

A total of 8458 of 29389 Nr-annotated unigenes were annotated in the COG database. Among 25 COG categories, only the general function prediction (2313) accounts for the largest proportion, followed by replication recombination and repair (1108), as well as transcription (1068). About 3.97% (336) unigenes present unknown function (Fig 4) and regarded as unique genes of licorice.

**Fig 5:**
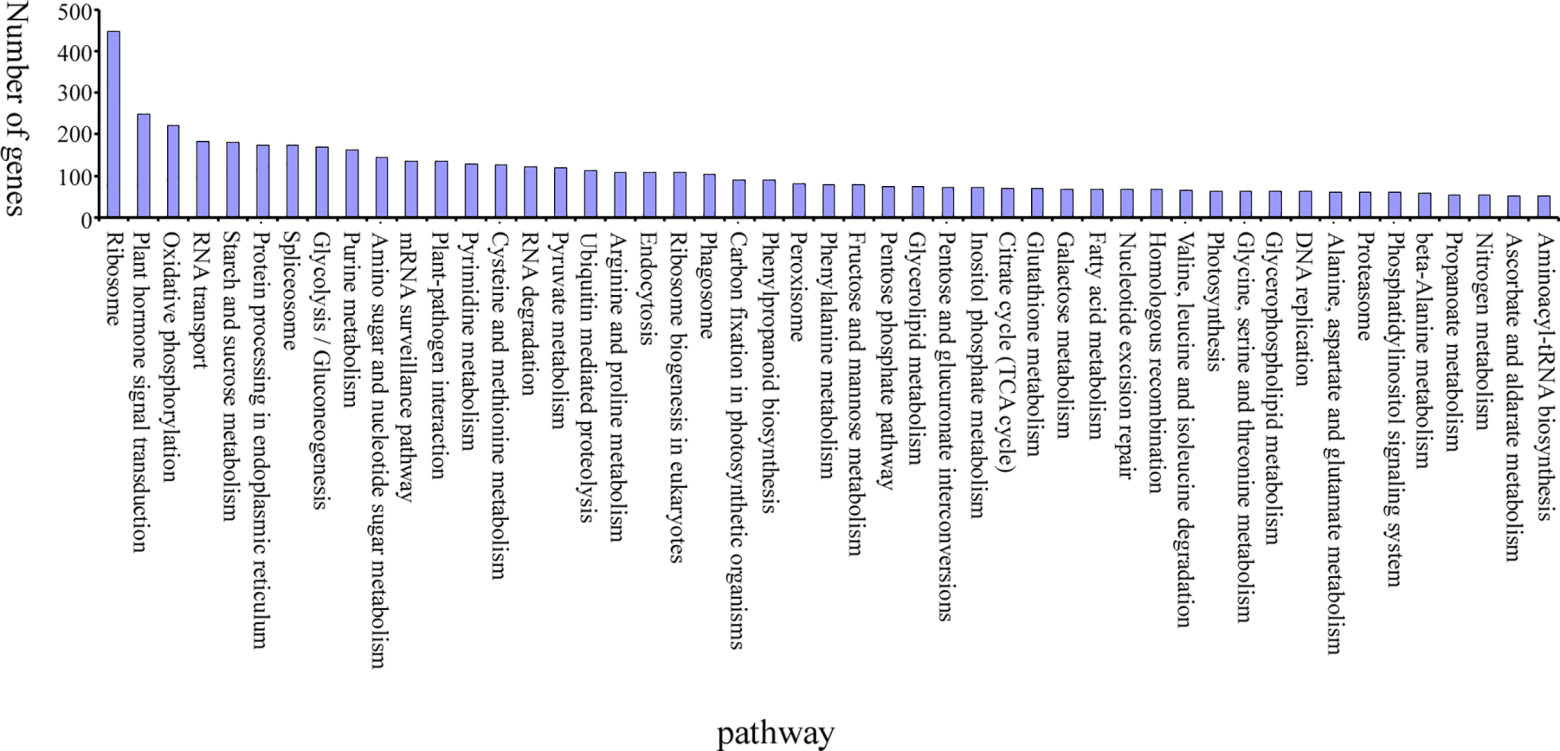
Distribution of pathway based on the KEGG database.

The KEGG database is employed to analyze gene products in the metabolic pathway of cells and determine their functions. About 6451 unigenes are in contrast with the KEGG database, of which 178 are involved in metabolic pathways. Ribosome (448) contains the most number of unigenes, followed by the plant hormone signal transduction (248), oxidative phosphorylation (221), RNA transport (182), and starch and sucrose metabolism (180). The distribution of pathway containing more than 50 genes, based on the KEGG database, is shown in Fig 5.

### 3. Main metabolism-related genes of licorice

Pharmacological research on licorice has mainly focused in glycyrrhizic and glycyrrhetinic acids, flavonoids, and polysaccharides [13]. Glycyrrhizic acid is an oleanane-type pentacyclic triterpene compound synthesized from mevalonic acid (MVA). Three molecules of acetyl COA condensation form 3-hydroxy-3-methyl glutaric acid-COA, resulting in the formation of MVA under the catalysis of HMG-CoA reductase. Focal phosphorylation, decarboxylation, and dehydration of the compound generate isopenteny diphosphate (IPP), which is an isomer of dimethylally diphosphate (DMAPP). The combination of IPP and DMAPP forms geranyl pyrophosphate (GPP), whereas the combination of GPP and IPP forms farnesyl diphosphate (FPP). The connected end-to-end connection squalene, namely, 2,3-oxidized squalene, generates β-amyrin under the action of the β-AS enzyme. The triterpenoid skeleton is then formed under the action of triterpenoid cyclase and through a series of reactions, such as adding oxygen to form glycyrrhizic acid [14]. Analysis of the KEGG pathways showed that 40 kinds of enzymes are involved in the terpenoid backbone biosynthesis. Moreover, annotation of 29,614 genes revealed that 16 genes participate in the synthesis of 11 kinds of enzymes, such as β-amyrin (Table 2). Nevertheless, the corresponding gene sequences of nine types of enzymes were not found. The genes corresponding to the biosynthesis of IPP and DMPPEC1.1.1.88, EC2.7.4.2, EC1.1.1.267, EC2.7.7.60, and EC5.3.3.2 were also not found. The corresponding genes of the other synthesized GPP, FPP, squalene, 2,3-oxidized squalene, and β-amyrin EC2.5.1.1, EC2.5.1.10, EC2.5.1.21, EC1.14.99.7 and 5.4.99 were also not detected.

**Table 2:** Enzymes related to glycyrrhizic acid metabolic pathways.

EC	Enzyme name	Gene
EC2.3.1.9	acetyl-CoA C-acetyltransferase	c12403.graph_c0 & c35153.graph_c0
EC2.3.3.10	hydroxymethylglutaryl-CoA synthase	c30007.graph_c0
EC1.1.1.34	hmg-CoA reductase	c33876.graph_c0 & c19615.graph_c0 & c21217.graph_c0
EC1.1.1.88	hydroxymethylglutaryl-CoA reductase	0
EC2.7.1.36	mevalonate kinase	c29677.graph_c0
EC2.7.4.2	phosphomevalonate kinase	0
EC4.1.1.33	diphosphomevalonate decarboxylase	c25954.graph_c0
EC2.2.1.7	1-deoxyxylulose-5-phosphate synthase	c31138.graph_c0 & c34113.graph_c0
EC1.1.1.267	DXP reductiosomerase	0
EC2.7.7.60	MEP cytidylytransferase	0
EC2.7.1.148	4-diphosphocytidyl-2-C-methyl-d-erythritol kinase	c27328.graph_c0
EC4.6.1.12	2-C-methyl-D-erythritol 2,4-cyclodiphosphate synthase	c20723.graph_c0
EC1.17.7.1	4-hydroxy-3-methylbut-2-enyl-diphosphate synthase	0
EC1.17.1.2	1-hydroxy-2-methyl-2-(E)-butenyl 4-diphosphate reductase	c26327.graph_c0
EC2.5.1.10	farnesyl diphosphate synthase	0
EC2.5.1.1	dimethylallyl transtransferase	0
EC2.5.1.21	farnesyl-diphosphate farnesyltransferase	c16912.graph_c0
EC1.14.99.7	squalene monooxygenase	c37309.graph_c0 & c5057.graph_c0
EC5.4.99.-	β-amyrin synthase	0
EC5.3.3.2	isopentenyl-PP isomerase	0

### 4. Development and SSR locus analysis

A total of 11,702 unigenes with a length of more than 1 Kb were found in the licorice transcriptome. Unigenes present a total length of 22,739,272 bp. To develop new molecular markers, we used the MISA software (http://pgrc.ipk-gatersleben.de/misa/misa.html) to determine potential microsatellites defined as mono- to hexa-nucleotide motifs. A total of 7,032 potential SSR loci were detected. The frequency of SSRs is 60.10%, and the average distribution distance is 3,234 bp. The SSR loci in unigenes are 48,611,547. Each unigene contained more than one SSR loci. The SSR locus numbers of mono-, di-, tri-, tetra-, penta-, and hexa-nucleotide repeats are 3,394, 1,692, 1,814, 101, 19, and 12, respectively (Table 3).

**Table 3:** Numbers of SSR repeat types in licorice.

Repeat type	Number
Mono-nucleotide	3394
Di-nucleotide	1692
Tri-nucleotide	1814
Tetra-nucleotide	101
Penta-nucleotide	19
Hexa-nucleotide	12
All of SSR locus number	7032
Number of sequence contain SSR loci	4861
Number of sequence contain more than SSR loci	1547
Total number of sequences examined	11702
Total size of examined sequences (bp)	22739272
Total number of identified SSRs	7032
Number of SSR containing sequences	4861
Number of SSRs present in compound formation	595

To identify licorice SSRs, we analyzed 66 kinds of repeat primitives (Table 4). Mono-, di-, tri-, tetra-, penta-, and hexa-nucleotide repeat motifs present 2, 4, 10, 21, 17, and 12 types, respectively. From the frequency of occurrence of different repeat primitives, the most abundant type is A/T, accounting for 46.23% of the total SSRs, followed by AG/CT (15.84%) and AAG/CTT (6.23%) repeats. Among the di-nucleotide repeat primitive, AG/CT appears frequently, accounting for 65.84% of the di-nucleotide SSR. In the tri-nucleotide repeat primitive, AAG/CTT appears frequently, accounting for 24% of tri-nucleotide SSR.

**Table 4:** Amounts of different SSR repeat motifs in licorice.

Motif	Repeat number	Frequency (%)
A/T	3251	46.23
C/G	143	2.03
AC/GT	339	4.82
AG/CT	1114	15.84
AT/AT	238	3.38
CG/CG	1	0.01
AAC/GGT	320	4.55
AAG/CTT	438	6.23
AAT/ATT	187	2.66
ACC/GTT	194	2.76
ACG/CGT	36	0.51
ACT/AGT	35	0.50
AGC/CTG	161	2.29
AGG/CCT	181	2.57
ATC/ATG	198	2.82
CCG/CGG	64	0.91
Others	132	1.88
Total	7032	100

**Fig 6:**
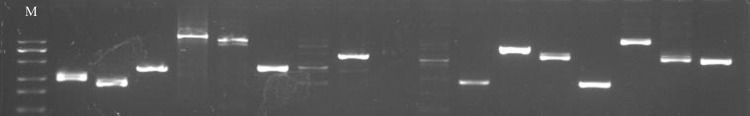
Photograph of PCR amplification results for SSR markers in licorice. The first line is the DNA ladder. The subsequent lines are the PCR products generated using different primers.

A total of 1,681 SSR sites were randomly selected from the SSR-containing sequences to design SSR primers with the Primer 6.0 software. Sixty-nine SSR pairs were randomly designed and synthesized. Sixty-four pairs were successfully used for PCR amplification of genomic DNA (Fig 6), whereas the five remaining pairs failed to generate PCR products at the same annealing temperatures. 53 pairs PCR products present the expected sizes and 11 pairs PCR products are larger than the expected sizes, which could be due to the fact that the PCR products contain introns. And 64 primer pairs polymorphic were detected in *Glycyrrhiza uralensis* Fisch., *Glycyrrhiza inflata* Bat., *Glycyrrhiza glabra* L. and *Glycyrrhiza pallidiflora Maxim*. by the 2.5% agarose electrophoresis analysis, the results showed that 33 pairs of primers were polymorphism in different species.

## Discussion

### 1. Licorice RNA-seq technology

Many technologies have been used to analyze and quantify the transcriptome of model or non-model organisms, such as *Arabidopsis*, rice, radish (*Raphanus sativus* L.), and *Haloxylon ammodendron*; as such, these techniques are vital to elucidate the complexity of growth and development of organisms. For medicinal plants, organ formation and development are controlled by complex interactions among genetic and environmental factors. The transcriptome data in publicly available libraries are insufficient and limited to describe the complex mechanisms of gene expression, as well as the genetic characteristics of species. Therefore, new generation of high-throughput sequencing technologies has been used as a powerful and cost-efficient tool for research on non-model organisms [9, 15]. In this experiment, we used RNA-Seq technology and obtained 5.41 Gb of clean data and 46, 641 unigenes from the assembly of the clean data (Table 1). The N50 length of the unigenes is 1,395 nt, with an average length of 787.40 nt. The results are comparable with the obtained unigenes in the recently published transcriptome analyses of other plant species, such as *H*. *ammodendron* (N50 = 1,354 bp, average length = 728 bp) [15], *Reaumuria soongorica* (N50 = 1,109 bp, average length = 677 bp) [16], and radish (*Raphanus sativus* L.) (N50 = 1,256 bp, average length = 820 bp) [17]. Longer unigenes may be obtained because of the developed Trinity software, which is a powerful software package for *de novo* assembly and generates increased number of full-length transcripts [11].

In this study, 29,614 unigenes were functionally annotated, whereas 17,027 (36.51%) did not obtain functional annotations. Unigenes may contain known functions of protein sequences because they are relatively short and lack conservative functional sequence. Known genes were not matched because their sequences contain missing parts and are relatively short. Moreover, unigenes contain non-coding RNA. In this regard, sequences were not functionally annotated because of insufficient number of unigenes and limited public information database.

### 2. Licorice genes related to the isoprenoid biosynthesis pathway

The isoprenoid biosynthesis pathway can synthesize kinetin, gibberellic acid, carotenoid, chlorophyll, sterols, monoterpenes, terpenes, and dolichol secondary metabolites [18]. The triterpene compound of glycyrrhizic acid is synthesized in the isoprene metabolic pathway. In this study, through transcriptome sequencing and KEGG database annotation, 40 kinds of enzymes are involved in terpenoid backbone biosynthesis, and 16 genes participate in the mevalonate pathway synthesis; moreover, 11 enzymes are coded in 29,614 annotated genes of licorice. Nevertheless, nine genes related to enzyme synthesis were not found. Li [5] studied the gene expression of wild licorice for 5 years and found 18 kinds of enzyme involved in licorice saponin synthesis; of these enzymes, 16 participate in the MVA synthesis of mevalonate kinase (EC2.7.1.36) and MEP synthesis pathway DXP synthase (EC2.2.1.7), whereas two enzymes are not related to the annotated genes. This result is significantly related to the sequencing of the samples with different ages and periods; transcriptome sequencing also show the different periods at which sample genes are expressed [19]. Nine kinds of enzyme in the synthesis of licorice saponins were not found. In different periods, the content of glycyrrhizin differs but the conclusion remains controversial. Liu [20] found that cultivated licorice with different ages presents varied contents of glycyrrhizin, total flavones, and polysaccharides; moreover, the highest content of glycyrrhizin was observed in the third year of cultivation. Sun [19] showed that the content of glycyrrhizin was higher after 4 years of cultivation. In addition, the quantity of synthesized saponin differs between the wild and cultivated *Glycyrrhiza*; licorice grows faster under cultivated conditions than that under wild conditions, resulting in higher primary metabolite contents. Although secondary metabolites are major components of traditional Chinese medicinal materials, their accumulation is related to adversity stress and are thus beneficial for accumulation of licorice saponin [5]. Therefore, we aim to design and conduct detailed tests and analyses by using different periods and ages of licorice material in the future.

### 3. Characteristics of SSR molecular markers

In the analysis of the SSR polymorphism loci of the licorice transcriptome with more than 1 Kb length and 11,702 unigenes by using the MISA software, a total of 7,032 SSR loci were detected with a frequency of 60.10% and an average distribution distance of 3,234 bp. In the licorice SSR loci, the most frequent repeat type is mono-nucleotide with 3,394 (48.27%), followed by tri- and di-nucleotide repeats, with 1,814 (25.80%) and 1,692 (24.06%), respectively. This distribution frequency differs from those of most plant genomes, such as field pea, faba bean, and autotetraploid *Alfalfa*, in which the most abundant repeat motif is tri-nucleotide (57.7%, 61.7%, and 61.19%, respectively) [10, 21]; in *Sesamum*, the most abundant repeat motif is di-nucleotide repeat motifs [22]. Autotetraploid *Alfalfa*, field pea, faba bean, and *P*. *cuspidatum* do not have mononucleotide repeat sequences, which could be due to the different standards used in SSR search criteria [9, 10, 21, 22]. In this study, we explored the mono-nucleotide repeat motifs in licorice; during the process, a condition where the mono-nucleotide repeat is dominant was generated, which decreases the number of other nucleotide repeats.

In this study, the occurrence frequency of tri-nucleotide repeats (25.80%) is higher than the di-nucleotide repeat frequencies (24.06%). Studies on *P*. *cuspidatum* [9], autotetraploid *Alfalfa* [10], *Asteraceae* (*Mikania micrantha*) [23], *Asteraceae* (*Chrysanthemum nankingense*) [24], and radish (*R*. *sativus* L.) [17] demonstrated similar conclusion. The di-nucleotide repeats of other plants, namely, rubber tree [25], *Sesamum* [22], and blunt snout bream [26], have higher frequencies than tri-nucleotide repeat frequencies. This finding may be due to the different genetics of different species and standards used for SSR search. In licorice di-nucleotide repeat motifs, AG/CT appeared the most, accounting for 15.84% of SSR. This result is consistent with that in *Sesamum* [22] and radish [17]. In plants, the presence of CT repeat sequence to 5′UTRs is probably related to reverse transcription and has a significant role in gene regulation [27]. By contrast, in the licorice tri-nucleotide repeat motifs, AAG /CTT appeared most, accounting for 6.23% of the total SSR. This result is consistent with that in *Sesamum* [22], and radish [17]; conversely, in rubber tree [25] and *Asteraceae* (*C*. *nankingense*) [24], AAG/TTC and CCA/GGT were the most abundant, respectively, which could be due to the frequency used in different encoding proteins of species.

Among 69 primer pairs, 64 (92.75%) were amplified successfully. The PCR success rate was similar to that in *Sesamum* [22], lower than that in rubber tree [25], and higher than that reported in a previous study [10]. These results suggest that the quality of assembled unigenes were high, and SSRs identified in our study could be used for future analysis.

## Supporting Information

S1 Table Sequences of 69 primer pairs for SSR markers. (XLS)
